# Association of rs4588 polymorphism in vitamin D binding protein gene with polycystic ovarian syndrome in Iranian women: a case-control study

**DOI:** 10.1186/s13104-024-06857-x

**Published:** 2024-07-27

**Authors:** Leila Nazarpoor Akbari, Asma Kheirollahi, Akram Vatannejad, Hediyeh Hamidi

**Affiliations:** Department of Comparative Biosciences, Faculty of Veterinary Medicine, University of Tehran, Tehran, Iran

**Keywords:** Vitamin D binding protein, Polycystic ovary syndrome, Polymorphism, Infertility, Recurrent pregnancy loss

## Abstract

**Objective:**

Vitamin D deficiency and variations in the vitamin D binding protein (VDBP) gene may play a role in the development of Polycystic ovary syndrome (PCOS). This study aims to investigate the association of the rs4588 polymorphism with PCOS in Iranian women, as well as its association with infertility and recurrent pregnancy loss (RPL) in these patients.

**Results:**

The analysis revealed statistically significant differences in the distributions of genotypes and alleles of the rs4588 polymorphism among the three groups (*p* < 0.0001). The AC genotype and A allele showed an association with an elevated risk of PCOS and infertility. In this study, no association was found between genotypes and alleles of the rs4588 polymorphism and the risk of RPL in women with PCOS. Subjects with the AA or AC genotype exhibited significantly higher levels of LDL compared to those with the CC genotype.

## Introduction

Polycystic ovary syndrome (PCOS), a common hormonal disorder affecting women of childbearing age, is characterized by irregular periods, excess androgen levels, and the presence of polycystic ovaries [1]. Common symptoms of PCOS include menstrual irregularities, hirsutism, acne, and weight gain [2]. The diagnosis of PCOS is established using the Rotterdam criteria, which require the presence of two out of three specific findings: oligomenorrhea or amenorrhea, clinical or biochemical hyperandrogenism, and polycystic ovaries [3]. PCOS is associated with a spectrum of severe health complications, encompassing infertility [4], recurrent pregnancy loss (RPL) [5], diabetes [6], cardiovascular disease [7], endometrial cancer [8], sleep apnea [9], depression [10], and anxiety [11]. The prevalence of infertility and RPL among women with PCOS is reported as more than 70% [12] and 30% [13], respectively.

Although the exact cause of PCOS is unknown, it may be due to a combination of genetic and environmental factors such as prenatal exposures to high levels of androgens or insulin, and diet and lifestyle habits [2]. Recent findings have established an association between vitamin D deficiency and the emergence of PCOS [14, 15]. Previous studies have consistently highlighted the prevalence of vitamin D deficiency in women diagnosed with PCOS [16, 17]. Additionally, studies have reported that vitamin D supplementation may ameliorate PCOS symptoms and enhance the likelihood of achieving pregnancy [18, 19].

Vitamin D-binding protein (VDBP), also known as GC, plays a critical role in the transport and metabolism of vitamin D [20]. VDBP is produced in the liver and circulates in the blood, where it binds to vitamin D and transports it to target tissues [21]. Several studies have shown that VDBP levels are associated with serum vitamin D levels [22, 23]. The VDBP gene contains various polymorphisms that can influence multiple aspects of the VDBP protein, encompassing its structure, function, and interactions with other molecules [24]. The rs4588 polymorphism is located within the gene region of VDBP on chromosome 4. It involves a single nucleotide change from cytosine (C) to adenine (A) at position 4588 in exon 11 of the VDBP gene [25]. This polymorphism has been associated with variations in circulating levels of vitamin D binding protein and subsequently affects the bioavailability and metabolism of vitamin D [25]. The variant allele (A) at this locus results in a VDBP variant with lysine (K) instead of threonine (T) at position 436. This substitution affects glycosylation, as the variant lacks O-glycosylation. The absence of glycosylation leads to lower VDBP levels, likely due to rapid clearance, and reduced binding affinity for 25-hydroxyvitamin D. Consequently, individuals with this variant have lower serum concentrations of VDBP and vitamin D, contributing to vitamin D deficiency [26–28].

While various studies have explored the association of the rs4588 polymorphism with diabetes, multiple sclerosis [29], obesity [30], pulmonary obstruction [31], asthma [32], and acute myeloid leukemia [33], limited research has addressed the association between this polymorphism and PCOS [34, 35]. To date, no study addressed the frequency of rs4588 in Iranian women with PCOS. Furthermore, to the best of our knowledge, no previous investigation has examined the association of rs4588 genotypes with infertility, as well as RPL, in women with PCOS. This study focused on investigating the association of rs4588 polymorphism with PCOS in Iranian women, as well as its relationship with infertility and RPL in Iranian women with PCOS.

## Materials and methods

### Study population

The participants of this study were sourced from the Obstetrics and Gynecology Department of the Ibn Sina Infertility Center in Tehran, Iran. Control subjects were specifically recruited from individuals undergoing routine checkups at the laboratory within the same center. This recruitment period spanned from May 2017 to January 2018. A statistically sound formula, informed by the Hajian-Tilaki study, was employed to determine the appropriate sample size [36]. The calculated sample size was 304; however, the number of participants analyzed was slightly lower due to missing individuals. All subjects were between 20 and 40 years of age. Subjects in this study were initially divided into two groups: 100 healthy fertile women and 197 women with PCOS. Women with PCOS were then further divided into two subgroups: infertile women (*n* = 96) and women with RPL (*n* = 101). PCOS patients were identified following the Rotterdam criteria [3]. Infertile women were defined as those who, despite participating in consistent unprotected sexual intercourse for 12 months or more, were unable to attain pregnancy [37]. RPL was defined as two or more consecutive miscarriages before the 20th week of pregnancy [38]. All procedures performed in studies involving human participants were by the ethical standards of the institutional and/or national research committee and with the 1975 Helsinki declaration as revised in 2008. The present research was approved by the Ethics Committee of Ibn Sina Infertility Center (IR.ACER.Avicenna.Res.1395.6). Informed consent was obtained using a written informed consent form before to beginning the study.

Women with Cushing’s syndrome, thyroid disorders, hyperprolactinemia, pituitary gland diseases, androgen-secreting tumors, or those who had used hormonal medications or steroids that induced insulin resistance at least 6 months before the study were excluded. It should be noted that, at the time of data collection, the women had not received any treatment, and blood samples were obtained before to the beginning of treatment during the follicular phase.

### Laboratory analysis

Following an overnight fast of at least 8 h, 10 mL of blood was collected from the individuals and dispatched to the laboratory for the measurement of the biochemical and hormonal parameters including age, BMI (body mass index), FBS (fasting blood sugar), insulin, HOMA-IR (Homeostatic Model Assessment for Insulin Resistance), TG (triglyceride), TC (total cholesterol), LDL (low-density lipoprotein), HDL (high-density lipoprotein), Free-T (free testosterone), LH (luteinizing hormone) and FSH (follicle stimulating hormone. These parameters were measured according to previously reported methods [39–41].

### Genotyping

DNA was extracted from peripheral blood leukocytes using a DNA extraction kit (Sina Clon, Iran). The extracted DNA was then amplified using PCR, with primers already described in the literature [42]. According to the protocol, 0.5 µL of forward primer (5’AAATAATGAGCAAATGAAAGAAGAC3’) and 0.5 µL of reverse primer (5’CAATAACAGCAAAGAAATGAGTAG3’) were combined with 1 µL of the extracted DNA, 10 µL of master mix, and 8 µL of deionized water. This mixture was then subjected to the following thermal cycler program: 1 cycle for 10 min at 94 °C, followed by 35 cycles at 94 °C for 30 s, 56 °C for 30 s, and 72 °C for 30 s, and a final cycle at 72 °C for 5 min. Finally, the PCR product with a size of 483 base pairs was visualized on a 1.2% agarose gel.

**Fig. 1:**
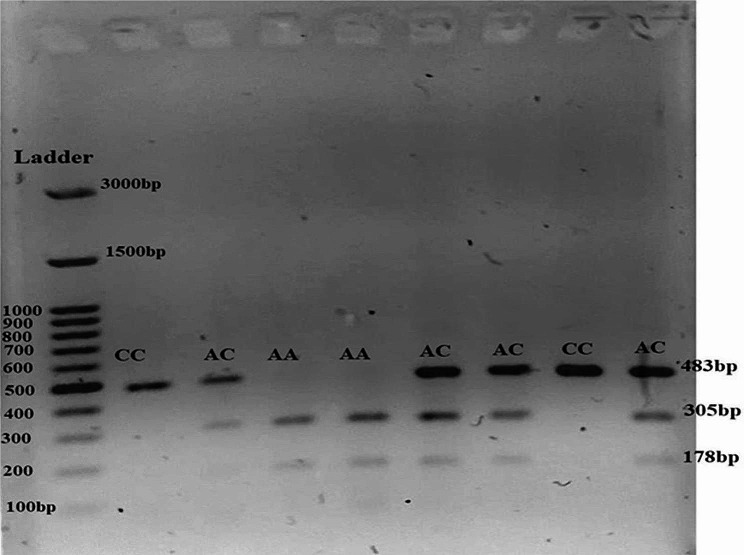
Genotype analysis of the VDBP rs4588 SNP using PCR-RFLP method. Homozygote CC (483 bp), homozygote AA (305 and 178 bp), and heterozygote AC (483, 305 and 178 bp) were visualized using the Syber safe stain on agarose 2%

After the amplification step, the PCR product was treated with the StyI restriction enzyme (Thermo Scientific) at 37 °C for 2 h and subsequently separated on a 2% agarose gel. The StyI restriction enzyme recognizes sites with the sequence C^CWWGG. PCR product of subjects with the C allele was not digested by the enzyme and shows a single band (483 bp). PCR products carrying the A allele were cleaved by the enzyme, resulting in two distinct bands (305 bp, 178 bp) (Fig. 1). In order to interpret our results precisely and differentiate between incomplete digestion and actual uncut variants, we incorporated several control measures. These controls included a sample undergoing full digestion with a longer enzyme incubation period, a negative control without any enzymes, and a positive control with a known rs4588 genotype.

### Statistical analysis

In this study, SPSS 26 software was used for statistical analysis. Variables that followed a normal distribution were reported in terms of mean and standard deviation, while variables with non-normal distribution were reported as median and IQR. The Shapiro-Wilk test was used to check the distribution of the data. For comparison between the two groups of patients and healthy individuals (PCOS and non-PCOS), the independent t-test was used for normally distributed data, and the Mann-Whitney test was used for non-normally distributed data. When comparing subgroups (PCOS-infertile, PCOS-RPL, and non-PCOS), one-way ANOVA and Bonferroni Post Hoc tests were used for normally distributed variables, and the Kruskal-Wallis test was used for non-normally distributed variables. The Hardy–Weinberg equilibrium for the genotype frequencies of the rs4588 polymorphism was examined. The chi-square test was utilized to analyze the frequency of genotypes and alleles among studied groups. Logistic regression analysis was employed to investigate the association between genotypes and alleles with PCOS. Similarly, multinomial regression analysis was used to explore the relationship between genotypes and alleles with infertility and RPL. A significance level of *P* < 0.05 was considered statistically significant.

## Results

The demographic and clinical data of the study participants are shown in Table 1. As previously reported [39–41], there was a significant difference in terms of age, insulin, HOMA-IR, HDL, FT, and FSH levels when comparing PCOS and related subgroups with the non-PCOS group. In the PCOS-RPL subgroup, the concentration of TG (136.71 ± 52.04) exhibited a notable increase compared to both the non-PCOS (118.70 ± 38.35) and PCOS-infertile subgroups (118.51 ± 57.33) (*p* = 0.01). In addition, infertile women with PCOS exhibited significantly higher levels of LH compared to the PCOS-RPL subgroup.

**Table 1:** Demographic and clinical data of study participants

	non-PCOS Mean ± SD (*n* = 100)	PCOS Mean ± SD (*n* = 197)	*P*-value^†^	PCOS-infertile Mean ± SD (*n* = 96)	PCOS-RPL Mean ± SD (*n* = 101)	*P*-value^‡^
Age (year)	33.37 ± 4.61 ^a, b^	30.24 ± 4.59	< 0.0001	30.3 ± 4.82^a^	30.17 ± 4.36^b^	< 0.0001
BMI (kg/m^2^)	25.73 ± 4.30	26.74 ± 4.76	0.08	27.02 ± 4.51	26.46 ± 5	0.1
FBS (mg/dL)	89 (82,96.5)	87 (83,93.5)	0.1	87.75 (83,93)	87 (82,94)	0.2
Insulin (µU/mL)	2.88 (1.96,4.3) ^a, b^	6.4 (2.85,8.1)	< 0.0001	5.1 (3.1,8.25)^a^	4.39 (2.51,7.92) ^b^	< 0.0001
HOMA-IR	0.59 (0.44,1.08) ^a, b^	1.02 (0.57,1.77)	< 0.0001	1.04 (0.65,1.75)^a^	1(0.54,1.8)^b^	< 0.0001
TG (mg/dL)	118.70 ± 38.35^b^	127.65 ± 55.36	0.1	118.51 ± 57.33^c^	136.71 ± 52.04 ^b, c^	0.01
TC (mg/dL)	168.62 ± 38.88	171.21 ± 35.95	0.5	169.91 ± 32.78	172.5 ± 38.96	0.7
LDL (mg/dL)	99.99 ± 29.42	97.5 ± 29.46	0.5	97.05 ± 27.73	97.95 ± 31.21	0.7
HDL (mg/dL)	46 (41,52) ^a, b^	43 (37.75,49)	0.006	43 (37,50) ^a^	44 (38,48) ^b^	0.02
Free_T (pg/mL)	1.48 (1.27,1.79)^& a,b^	3.25 (2.52, 3.96)	< 0.0001	3.1 (2.32, 3.7)^a^	3.29 (2.73,4.15)^b^	< 0.0001
LH (IU/L)	6.17 ± 2.31^&^	6.71 ± 3.92	0.9	7.81 ± 4.64^c^	5.6 ± 2.61^c^	< 0.0001
FSH (IU/L)	8.15 (6.39,9.6) ^a, b^	5.9 (4.35,7.42)	< 0.0001	6.02 (4.75,7.2) ^a^	5.9 (4.24,7.61) ^b^	< 0.0001

†Independent t test for comparison between PCOS and non-PCOS groups

‡One-way ANOVA with Bonferroni post hoc test for comparison among PCOS-infertile, PCOS-RPL and non-PCOS

^&^ LH and Free-T levels in 45 individuals have been measured in the non-PCOS group

Similar uppercase letters indicate significant differences among pairwise groups with Bonferroni’s approach

PCOS, Poly Cystic Ovary Syndrome; BMI, Body Mass Index; T, Testosterone; LH: luteinizing hormone; FSH: follicle-stimulating hormone

Genotypic and allelic frequencies of the rs4588 polymorphism were in Hardy-Weinberg equilibrium (*P* > 0.05). The results of the genotype and allele frequencies of rs4588 SNP are shown in Table 2. The minor allele frequency (MAF) for rs4588 in the total population was 29%. According to the results, there was a significant difference in the distribution of genotypes and alleles between PCOS and non-PCOS groups. Interestingly, the AC genotype significantly increased the risk of PCOS (OR: 2.37, 95% CI [1.48–3.94], *p* = 0.001) when compared to the CC genotype. Additionally, the A allele increased the risk of PCOS (OR: 1.98, 95% CI [1.22–3.23], *p* = 0.006). According to multinomial regression analysis, the AC genotype and the A allele were associated with an increased risk of infertility among PCOS patients (OR: 4.39, 95% CI [2.35–8.19], *p* < 0.0001, and OR: 3.66, 95% CI [1.99–6.72], *p* < 0.0001, respectively). However, no association was observed between the genotypes and alleles of rs4588 and the risk of RPL in women with PCOS.

**Table 2:** Genotypic and allelic distribution of rs4588 polymophysm in PCOS and non-PCOS groups

Genotype/ allele	Non- PCOS *N* (%)	PCOS *N* (%)	PCOS- infertile *N* (%)	PCOS- RPL *N* (%)	PCOS vs. Non- PCOS*		PCOS-infertile Vs. Non- PCOS**		PCOS-RPL Vs. Non-PCOS^**^	
					OR (CI)	*P*- value	OR (CI)	*P*-value	OR (CI)	*P*-value
CC	55 55%	75 38.1%	24 25%	51 50.5%	ref	-	ref	-	Ref	
AC	37 37%	120 60.9%	71 74%	49 48.5%	2.37 (1.43–3.94)	0.001	4.39 (2.35–8.19)	< 0.0001	1.42 (0.80–2.53)	0.222
AA	8 8%	2 1%	1 1%	1 1%	0.18 (0.03–0.89)	0.036	0.28 (0.03–2.41)	0.251	0.13 (0.01–1.11)	0.063
C	147 73.5%	270 68.52%	119 61.97%	151 74.75%	ref	-	ref		Ref	
A	53 26.5%	124 31.47%	73 38.02%	51 25.24%	1.98 (1.22–3.23)	0.006	3.66 (1.99–6.72)	< 0.0001	1.19 (0.68–2.08)	0.523

*The association between genotype and allele frequencies with PCOS was examined using logistic regression analysis

**The association between genotype and allele frequencies with infertility and RPL was investigated using multinomial regression analysis

The association between biochemical factors and rs4588 genotypes is shown in Table 3. According to the results, the LDL level was significantly higher in individuals with the AA + AC genotype compared to those with the CC genotype (*p* < 0.05). No significant differences were observed between the levels of other biochemical and hormonal factors and genotypes.

**Table 3:** Association of clinical characteristics with genotypic frequencies for VDBP genotypes in PCOS patients

Biochemical/hormonal parameters	CC (*n* = 75)	AA + AC (*n* = 122)	*P*-value
Age^#^(years)	31.39 ± 4.60	31.11 ± 4.81	0.624
BMI^#^(kg/m^2^**)**	26.52 ± 4.64	26.49 ± 4.15	0.945
FBS^#^(mg/dL)	89.79 ± 9.75	88.61 ± 8.80	0.284
Insulin* (µU/mL)	3.90 (2.41,6.72)	4.29 (2.50,7.20)	0.357
HOMA-IR^#^	1.05 ± 0.79	0.94 ± 0.64	0.340
TG^*^(mg/dL)	116 (93,145)	120 (85.25,160.75)	0.647
TC^#^(mg/dL)	167.04 ± 39.75	174.41 ± 35.93	0.101
LDL^*^(mg/dL)	92 (76,110)	99 (82,121.75)	0.035
HDL* (mg/dL)	44.50 (39,50)	44 (40,51)	0.861
HomoCys^*^(mmol/L)	10.90 (8,13.48)	10.51 (8.42,14.15)	0.857
Free_T^#^(pg/mL)	2.63 ± 1.43	2.89 ± 1.18	0.251
LH^*^(IU/L)	5.70 (3.71,7.93)	6.43 (4.45,8.65)	0.107
FSH^*^(IU/L)	6.60 (4.70,8.89)	6.10 (4.82,7.61)	0.275
Adiponectin^#^(µg/mL)	4.08 ± 2.37	3.58 ± 2.13	0.197
hsCRP^#^(mg/L)	3.47 ± 1.32	3.46 ± 1.19	0.948

#Parameters with a normal distribution were reported with mean and standard deviation and were compared using independent t-test

*Parameters with a non-normal distribution were reported with median and IQR and were compared using the Mann-Whitney test

## Discussion

Metabolic disorders are common complications in individuals with PCOS. While various clinical parameters showed significant differences between PCOS and non-PCOS groups, BMI, FBS, TC, and LDL levels did not exhibit significant variations between studied groups (Table 1). However, some studies reported significant differences in these parameters between PCOS and non-PCOS individuals [35, 43]. The lack of uniform findings across studies regarding these parameters may be influenced by factors such as ethnicity, age, and sample size. Elevated levels of HOMA-IR and insulin were observed in PCOS women, consistent with previous research [35, 44, 45]. Our results showed that TG levels were significantly higher in the PCOS-RPL subgroup compared to both the non-PCOS and PCOS-infertile subgroups (*p* = 0.01). In this context, Liu et al. demonstrated that elevated TG are associated with IR in patients with RPL [46]. Therefore, elevated TG levels may play a crucial role in the pathogenesis of RPL in PCOS patients through increased IR. The elevated levels of LH observed in infertile women could be attributed to its potential role in anovulation and, consequently, infertility [47]. The higher testosterone levels among PCOS patients are likely explained by the elevated androgen levels typically associated with this condition, a trend supported by existing literature [35, 48].

In this study, we investigated the frequency of the rs4588 polymorphism in Iranian women with PCOS, evaluating its association with both PCOS itself and PCOS-associated infertility and RPL. Our results demonstrated a significant association between the AC genotype and the A allele with PCOS (*p* = 0.001 and 0.006, respectively) and infertility (*p* < 0.0001) in women with PCOS. However, no significant association was found between the genotypes or alleles of this polymorphism and RPL in women with PCOS (Table 2).

Nowadays, the deficiency of vitamin D is recognized as one of the contributing factors to PCOS. In this context, Haldar et, al. reported that vitamin D deficiency could be considered as an initial factor in the onset and progression of PCOS [34]. It has been revealed that supplementation of vitamin D in women with PCOS-related infertility resulted in a reduction in body mass index, improved follicular maturation, regular menstruation, and improvement in hyperandrogenism [49]. However, some studies haven’t observed a significant correlation between vitamin D supplementation and improved insulin resistance or increased sensitivity [50].

Given the variable response to vitamin D supplementation, the potential involvement of genetic factors, especially polymorphisms, in determining serum levels of vitamin D is highlighted. Various studies have addressed the association of the rs4588 polymorphism with serum levels of vitamin D. Lafi et al. (2015) investigated this association in 381 individuals in Jordan and found that genotypes AA and AC of rs4588 were associated with an increased risk of vitamin D deficiency in healthy individuals [51]. Other studies have shown that individuals with the CC genotype had higher levels of vitamin D compared to those with the AA genotype [52] and that individuals with the A allele had lower levels [53]. Several other studies also confirm the association of the A allele from the rs4588 polymorphism with vitamin D deficiency [54–57].

Considering the association of the rs4588 polymorphism with reduced vitamin D levels, various research studies have investigated its association with a variety of diseases, including tuberculosis, asthma, and metabolic syndrome [25, 32, 52, 58]. In this context, few studies have investigated the association between the rs4588 polymorphism and PCOS [34, 35].

A study conducted in Korea involving 432 women with PCOS and 927 healthy women found no significant association between VDBP polymorphisms and PCOS [35]. Similarly, a study in southern Brazil with 291 women (191 with PCOS and 100 in the control group) observed no association between the rs4588 and rs7041 polymorphisms and PCOS [44]. The findings of the study by Haldar on 50 Indian women with PCOS and 50 healthy women indicated that the allelic combination (GC1F/1F: T allele of rs4588 and C allele of rs7041) of the VDBP increased the risk of PCOS in vitamin D deficient women [34]. In contrast, our study found that the AC genotype and A allele significantly increase the risk of PCOS in women (*p* = 0.001 and 0.006, respectively). This discrepancy can be justified by considering racial differences and variations in the study population size. Given the association of the A allele with reduced vitamin D levels, the rs4588 polymorphism may play a role in the pathogenesis of PCOS by influencing vitamin D levels.

The 1000 Genomes Project Phase 3 data reports an MAF of 20% for rs4588 in the combined population. However, our study observed a higher MAF of 29%. This discrepancy highlights the population-specific variability in MAF for rs4588.

In this study, we investigated the association of the rs4588 polymorphism with infertility and RPL in women with PCOS for the first time. Chi-square analysis of data revealed a significantly higher frequency of the AC genotype (74%) and the A allele (38%) in the infertile PCOS group compared to the non-PCOS control group (37% and 26.5%, respectively). In contrast, frequencies of the AC genotype and A allele in the RPL-PCOS group were relatively similar to the control group. Interestingly, the infertile PCOS group exhibited a significantly higher frequency of both the AC genotype (74%) and the A allele (38%) compared to the RPL-PCOS group (48.5% and 25.2%, respectively). Moreover, regression analysis indicated that the AC genotype and the A allele were associated with a 3.3-fold (*p* < 0.0001) and a 2.6-fold (*p* < 0.0001) increased risk of infertility in PCOS patients, respectively. However, no significant association was observed between these genotypes and alleles in the RPL-PCOS group. The prevalence of infertility in women with PCOS is reported 70 to 80% [59]. Since vitamin D plays a crucial role in the female reproductive process, vitamin D deficiency decreases the chances of successful fertilization [60]. Therefore, this polymorphism may be related to infertility in PCOS women through its negative effect on vitamin D levels.

Our study found that individuals with the AC or AA genotypes had higher LDL levels compared to those with the CC genotype (*p* = 0.035) (Table 3). Previous studies have not reported any association between rs4588 genotypes and elevated LDL levels in individuals with PCOS. However, Zhao et al. (2022) did report an association between the AA genotype of rs4588 and higher triglyceride levels and lower HDL levels in individuals with metabolic syndrome [61]. As previously noted, the rs4588 polymorphism has been associated with vitamin D deficiency. Given the link between vitamin D deficiency and dyslipidemia, various hypotheses exist regarding the regulatory role of vitamin D in lipid metabolism. Vitamin D functions to regulate lipid values by enhancing intestinal calcium absorption, thereby reducing intestinal fatty acid absorption and lowering cholesterol levels [62, 63]. Additionally, it inhibits parathyroid hormone (PTH), leading to reduced TG levels through increased lipolytic activity and modulation of lipoprotein metabolism, resulting in lower VLDL-C and higher HDL-C levels [64]. Moreover, vitamin D promotes the conversion of cholesterol into bile acids in the liver, further contributing to decreased cholesterol levels [64]. The rs4588 polymorphism may contribute to the development of obesity, increased body mass index, and an elevated risk of heart disease and metabolic syndrome in PCOS patients, possibly through its influence on lipids metabolism.

## Conclusions

This study identified a significant difference in the frequency of rs4588 genotypes in the PCOS group compared to the control group. The AC genotype and the A allele were associated with an increased risk of PCOS and PCOS-associated infertility. Considering the link between the allele A and decreased vitamin D levels, it seems that the rs4588 polymorphism may play a role in the pathogenesis of PCOS by influencing vitamin D levels. Nevertheless, further research is needed to elucidate the precise mechanisms by which the rs4588 polymorphism contributes to PCOS pathogenesis.

## Limitations

The study has limitations that should be considered. First, this study involved a relatively small group of Iranian women. To validate the findings and enhance generalizability, further research with a larger and more diverse population is necessary. Second, a notable limitation is the absence of serum vitamin D level measurements, which could provide valuable insights into the potential mechanisms underlying our observations.

## Data Availability

All data generated or analyzed during this study are included in this published article.

## Abbreviations

BMI: Body mass index
FSH: Follicle-Stimulating Hormone
FBS: Fasting blood sugar
Free-T: Free testosterone
IQR: Interquartile range
LH: Luteinizing hormone
LDL: Low-density lipoprotein
OR: Odds Ratio
PCOS: Polycystic Ovary Syndrome
PCR: Polymerase chain reaction
RFLP: Restriction fragment length polymorphism
RPL: Recurrent pregnancy loss
VDBP: Vitamin D Binding Protein
